# TransGEM: a molecule generation model based on Transformer with gene expression data

**DOI:** 10.1093/bioinformatics/btae189

**Published:** 2024-04-17

**Authors:** Yanguang Liu, Hailong Yu, Xinya Duan, Xiaomin Zhang, Ting Cheng, Feng Jiang, Hao Tang, Yao Ruan, Miao Zhang, Hongyu Zhang, Qingye Zhang

**Affiliations:** Hubei Key Laboratory of Agricultural Bioinformatics, College of Informatics, Huazhong Agricultural University, Wuhan 430070, P.R. China; Hubei Key Laboratory of Agricultural Bioinformatics, College of Informatics, Huazhong Agricultural University, Wuhan 430070, P.R. China; Hubei Key Laboratory of Agricultural Bioinformatics, College of Informatics, Huazhong Agricultural University, Wuhan 430070, P.R. China; Hubei Key Laboratory of Agricultural Bioinformatics, College of Informatics, Huazhong Agricultural University, Wuhan 430070, P.R. China; Hubei Key Laboratory of Agricultural Bioinformatics, College of Informatics, Huazhong Agricultural University, Wuhan 430070, P.R. China; Hubei Key Laboratory of Agricultural Bioinformatics, College of Informatics, Huazhong Agricultural University, Wuhan 430070, P.R. China; Hubei Key Laboratory of Agricultural Bioinformatics, College of Informatics, Huazhong Agricultural University, Wuhan 430070, P.R. China; Hubei Key Laboratory of Agricultural Bioinformatics, College of Informatics, Huazhong Agricultural University, Wuhan 430070, P.R. China; Hubei Key Laboratory of Agricultural Bioinformatics, College of Informatics, Huazhong Agricultural University, Wuhan 430070, P.R. China; Hubei Key Laboratory of Agricultural Bioinformatics, College of Informatics, Huazhong Agricultural University, Wuhan 430070, P.R. China; Hubei Key Laboratory of Agricultural Bioinformatics, College of Informatics, Huazhong Agricultural University, Wuhan 430070, P.R. China

## Abstract

**Motivation:**

It is difficult to generate new molecules with desirable bioactivity through ligand-based *de novo* drug design, and receptor-based *de novo* drug design is constrained by disease target information availability. The combination of artificial intelligence and phenotype-based *de novo* drug design can generate new bioactive molecules, independent from disease target information. Gene expression profiles can be used to characterize biological phenotypes. The Transformer model can be utilized to capture the associations between gene expression profiles and molecular structures due to its remarkable ability in processing contextual information.

**Results:**

We propose TransGEM (Transformer-based model from gene expression to molecules), which is a phenotype-based *de novo* drug design model. A specialized gene expression encoder is used to embed gene expression difference values between diseased cell lines and their corresponding normal tissue cells into TransGEM model. The results demonstrate that the TransGEM model can generate molecules with desirable evaluation metrics and property distributions. Case studies illustrate that TransGEM model can generate structurally novel molecules with good binding affinity to disease target proteins. The majority of genes with high attention scores obtained from TransGEM model are associated with the onset of the disease, indicating the potential of these genes as disease targets. Therefore, this study provides a new paradigm for *de novo* drug design, and it will promote phenotype-based drug discovery.

**Availability and implementation:**

The code is available at https://github.com/hzauzqy/TransGEM.

## 1 Introduction

Drug research and development (R&D) is an expensive, complex, long process with low success rate (Chan *et al.* 2019, Zhu 2020). Therefore, a multitude of artificial intelligence methods have been used to accelerate the drug R&D. *De novo* drug design plays a central role in the field of artificial intelligence (AI)-aided drug discovery by exploring chemical space in an automated way to generate new compounds (Jiménez-Luna *et al.* 2021, Pereira *et al.* 2021). The ligand-based *de novo* drug design only generates new molecules based on prior knowledge of given structural ligands (Wang *et al.* 2020, Xu *et al.* 2021). However, most of the generated molecules deviate from the expected properties and lack bioactivity. The receptor-based *de novo* drug design heavily depends on target protein information, particularly the structure information of the target protein (Lin *et al.* 2020, Grant and Sit 2021, Robson 2022). However, the target proteins of emerging diseases remain unidentified, and the target proteins of some complex diseases have not been determined or the structures have not been resolved. These diseases pose challenges for receptor-based *de novo* drug design approaches.

Early drug R&D predominantly focused on phenotypic drug discovery (PDD) (Vincent *et al.* 2022). PDD in combination with AI is becoming increasingly mature, and it has been accepted as a mode of drug discovery in academia and pharma (Vincent *et al.* 2022, Sadri 2023). Gene expression profiles can be used to characterize cell and biological phenotypes (Pham *et al.* 2021), and have been successfully used for *de novo* drug design. Méndez-Lucio *et al.* (2020) devised a conditional Generative Adversarial Network (GAN) framework using two discriminators to respectively assess the association between generated molecules with gene expression profiles and their authenticity. However, the proportion of valid molecules generated by this model was relatively low, only 8.2% (Méndez-Lucio *et al.* 2020). Furthermore, Adversarial Autoencoder (AAE) (Shayakhmetov *et al.* 2020), Variational Autoencoder (VAE) (Das *et al.* 2023, Pravalphruekul *et al.* 2023), and fragment-based generative model (Pham *et al.* 2022) have been used to generate new molecules from gene expression data. These above models generated a greater number of valid molecules than GAN, and these methods designed molecules based on gene knocked-out transcriptomic profiles. Nevertheless, molecules generated from these knock-out transcriptomic profiles exhibited a notable lack of correlation with known inhibitors of the targeted genes (Pham *et al.* 2022, Das *et al.* 2023, Pravalphruekul *et al.* 2023). PaccMann^RL^ (Born *et al.* 2021), a hybrid model combined VAE with reinforcement learning, was designed to generate molecules based on target-specific transcriptomic data. However, the final output molecules were optimized using the anticancer drug sensitivity prediction model within a reinforcement learning framework.

The cost-effective gene-expression experiments led to development of various open-source transcriptomics databases, such as the Gene Expression Omnibus (Clough and Barrett 2016), The Cancer Genome Atlas (Tomczak *et al.* 2015), Connectivity Map (Lamb *et al.* 2006), and Library of Integrated Network-based Cellular Signatures 1000 landmark genes (LINCS1000) (Subramanian *et al.* 2017). Among them, the LINCS1000 database has been used in multiple studies for molecular generation based on gene expression data (Pham *et al.* 2022, Das *et al.* 2023, Pravalphruekul *et al.* 2023). LINCS1000 database comprises gene expression data of various cell lines subjected to different perturbation conditions, including small molecule compound addition, gene knockout, gene overexpression, and other treatments (Subramanian *et al.* 2017). The LINCS1000 database comprises gene expression data at five levels of various perturbed cell lines, but only level 5 data have been used in the existing studies (Pham *et al.* 2022, Das *et al.* 2023, Pravalphruekul *et al.* 2023). However, level 5 data have been demonstrated to exhibit noise when considered as gene expression data (Pham *et al.* 2021). The creators of the LINCS 1000 database also indicate that the level 5 data are more suitable for biomedical discovery (Subramanian *et al.* 2017), such as identifying differentially expressed genes. Current several studies have attempted to mitigate noise in level 5 data, such as MODZ (Subramanian *et al.* 2017), Characteristic Direction (Clark *et al.* 2014, Duan *et al.* 2016) and DeepCE (Pham *et al.* 2021); however, the efficacy of these methods is not markedly significant.

To address the above issues, we propose a deep-learning framework and name it TransGEM (Transformer-based model from gene expression to molecules), which is a phenotype-based *de novo* drug design model. The TransGEM model can generate drug-like molecules with therapeutic potential for a specific disease solely based on disease-associated gene expression data. The model consists of gene expression encoder, molecule embedding layer, Transformer decoder, and generator. Furthermore, we used stable level 3 data in the LINCS dataset, as opposed to level 5 data with higher noise, to characterize gene expression information. Collectively, the primary contributions of our work are as follows.

1. A specialized embedding method for gene expression difference values is adopted, which is suitable and intuitive in characterizing changes in gene expression levels.
2. When generating molecules targeting specific disease, the TransGEM model allocates more attention to disease-related genes, and thus these genes may serve as potential targets for the disease.

## 2 Materials and methods

### 2.1 Model construction

**Figure 1.**
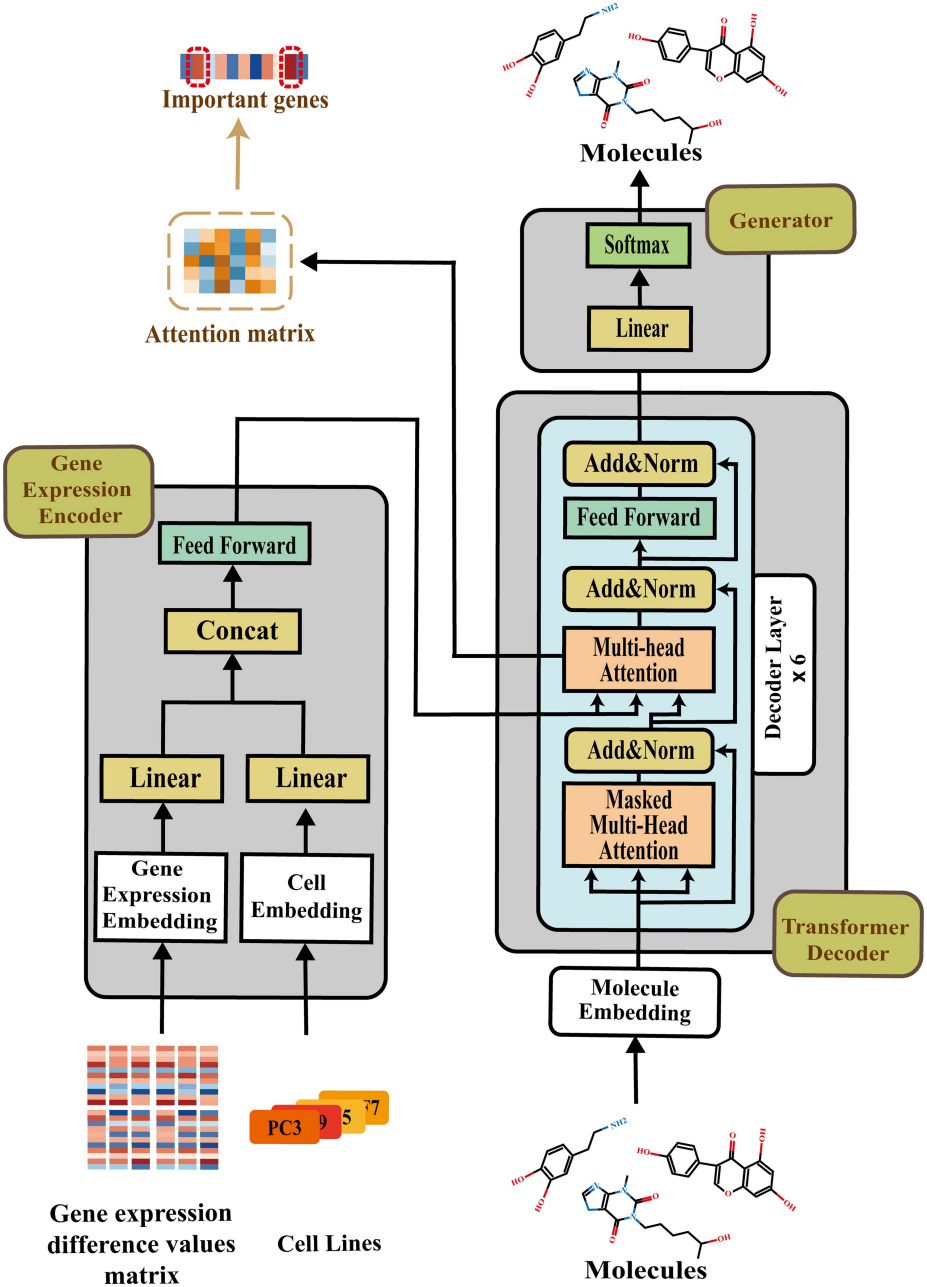
The workflow of TransGEM model. The model consists of Gene expression encoder, Transformer decoder, and Generator module. The gene expression embedding layer and cell embedding layer are used to embed gene expression differences and cell line types, respectively. Then, the embedding information of these two components were integrated and imported into the Transformer decoder module by the Gene expression encoder module. The molecular embedding layer is used to embed molecular representations, and its embedding information is then fed into the Transformer decoder module. The Transformer decoder module, including a total of six decoder layers, mainly used to learn the correlation between the gene expression encoder and the molecular embedding layer. Finally, the learning outcomes of the Transformer decoder were imported to the Generator module, and molecules that meet the rules are generated. In addition, during the molecular generation process, attention matrices can be extracted in the multi-head attention layer of the Transformer decoder module. Based on these attention matrices, important genes related to disease could be identified

This study proposes TransGEM model, an end-to-end molecule generation model based on gene expression data. The model consists of gene expression encoder, molecule embedding layer, Transformer decoder, and generator (Fig. 1). The cell line type and gene expression difference values before and after molecules perturbation are encoded by the gene expression encoder. These molecules are embedded by the molecule embedding layer. The above two pieces of information are inputted to the decoder of the Transformer model. The objective is to learn the association between a molecule and gene expression difference values specific to the cell line perturbed by this molecule through the decoder. Finally, molecules are reconstructed through the generator. More details about TransGEM components are described as follows.

#### 2.1.1 Gene expression encoder

The gene expression information comprises two components, G={C, E}, where C denotes cell line type and $E=(e_{1},\;e_{2},\cdot \cdot \cdot ,\;e_{k})$ represents the expression difference values of *k* genes between this cell line and normal tissue cells. The gene expression encoder is primarily used to separately embed two distinct sets of information, integrate them, and subsequently output the integrated information.

Initially, cell line type is embedded as one-hot vector, resulting in the following through a linear layer to update into $v_{c}\in R^{d}$:

(1) $$v_{c}=\mathrm{Linear}(\mathrm{One}-\mathrm{hot}(C))$$

where One-hot(*) represents one-hot encoding embedding, and Linear(*) denotes linear transformation.

**Figure 2.**
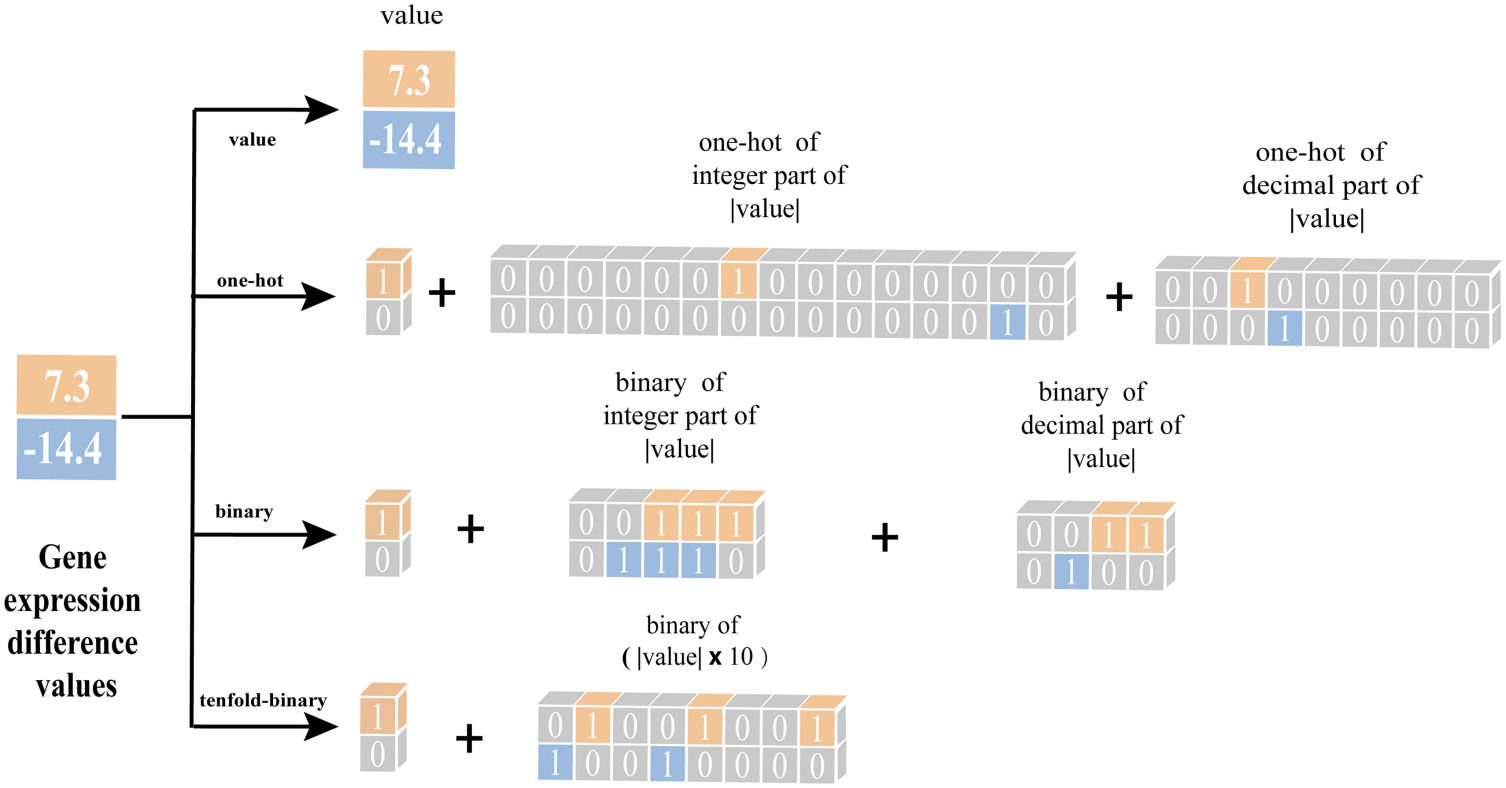
The four methods for embedding gene expression difference value. The value 7.3 and −14.4 were used as examples to illustrate. Value: directly use numerical values as input. One hot: the first dimension was used to represent positive and negative numbers, 1 represents positive numbers, and 0 represents negative numbers. The numerical value was decomposed into integer and decimal parts, each encoded separately with one-hot. Finally, these parts are directly connected together to express the gene expression difference value. Binary: except for the numerical value was decomposed into integer and decimal parts, each part being converted into binary and then embedded, the rest are the same as one hot type. Tenfold-binary: except for the numerical value was first multiplied by 10, then converted into binary and embedded, the rest are the same as one hot type

The gene expression difference values are embedded by $f_{\mathrm{emb}}$, resulting in the following through a linear layer to update into $v_{e}$:

(2) $$v_{e}=\mathrm{Linear}(f_{\mathrm{emb}}(E))$$

where $f_{\mathrm{emb}}$ (*) represents gene expression difference value embedding function. As illustrated in Fig. 2, this study explored 4 gene expression difference value embedding functions.

1. value:
  (3) $$f_{\mathrm{emb}}\left(e_{i}\right)=e_{i}$$
2. one-hot
  (4) emb1=1, if ei>00, if ei≤0
  (5) $${\mathrm{emb}}_{2}=\;\mathrm{One}-\mathrm{hot}\left(\left|\left\lfloor e_{i}\right\rfloor \right|\right)$$
  (6) $${\mathrm{emb}}_{3}=\;\mathrm{One}-\mathrm{hot}\left(\left(\left|e_{i}-\left\lfloor e_{i}\right\rfloor \right|\right)\times 10\right)$$
  (7) $$f_{\mathrm{emb}}\left(e_{i}\right)=\;\mathrm{Conact}({\mathrm{emb}}_{1},{\;\mathrm{emb}}_{2},\;{\;\mathrm{emb}}_{3})$$
3. binary
  (8) emb1=1, if ei>00, if ei≤0
  (9) $${\mathrm{emb}}_{2}=\;\mathrm{Pad}\left(\mathrm{Binary}\left(\left|\left\lfloor e_{i}\right\rfloor \right|\right),\;5\right)$$
  (10) $${\mathrm{emb}}_{3}=\;\mathrm{Pad}\left(\mathrm{Binary}\left(\left(\left|e_{i}-\left\lfloor e_{i}\right\rfloor \right|\right)\;\times \;10\right),\;4\right)$$
  (11) $$f_{\mathrm{emb}}\left(e_{i}\right)=\;\mathrm{Conact}({\mathrm{emb}}_{1},{\;\mathrm{emb}}_{2},\;{\;\mathrm{emb}}_{3})$$
4. 10-fold-binary
  (12) emb1=1, if ei > 00, if ei ≤ 0
  (13) $${\mathrm{emb}}_{2}=\;\mathrm{Pad}\left(\mathrm{Binary}\left(\left|e_{i}\right|\;\times \;10\right),\;9\right)$$
  (14) $$f_{\mathrm{emb}}\left(e_{i}\right)=\;\mathrm{Conact}({\mathrm{emb}}_{1},\;{\mathrm{emb}}_{2})$$

where $e_{i}$ denotes the i-th gene expression difference value in set E, $\mathrm{Concat}(v_{i},v_{j})$ represents concatenation of vectors $v_{i}$ and $v_{j}$, One-hot(*) and $\mathrm{Binary}\left(*\right)\;$signify one-hot and binary encoding embedding respectively, $\left\lfloor e_{i}\right\rfloor$ denotes the integer part of $e_{i}$, $\left|e_{i}\right|$ indicates the absolute value of $e_{i}$, and $\mathrm{Pad}(v_{i},\;n)$ signifies the dimensional padding of vector $v_{i}$ to achieve a dimensionality of n. Subsequently, the embedding vector $v_{c}$ of the cell line and the embedding vector $v_{e}$ of the gene expression difference value are concatenated. Following this, the concatenated vector undergoes a linear layer and a feedforward neural network, resulting in the encoded matrix $G_{0}$ for gene expression information. The feedforward neural network comprised two linear layers, with the initial layer using Rectified Linear Unit (ReLU) as its activation function.

(15) $$G_{0}=\mathrm{FNN}(\mathrm{Linear}(\mathrm{Concat}(v_{c},\;v_{e})))$$

where FNN(*) represents the feedforward neural network.

#### 2.1.2 Molecule embedding

In this study, molecules are represented as corresponding self-referencing embedded strings (SELFIES) (Krenn *et al.* 2020). Firstly, all unique SELFIES in the LINCS 1000 database are split into tokens, and these tokens are used to build a dictionary. Therefore, a given molecule M can be represented as a collection of *L* tokens, $M=(t_{1},\;t_{2},\cdot \cdot \cdot ,{\;t}_{L})$. Through the molecular embedding layer, the molecule *M* can be embedded to $M_{0}\;\in R^{L\times d}$, where *L* represents the number of tokens contained in molecule *M*, and *d* denotes the embedding dimension.

(16) $$M_{0}=\mathrm{Embed}\left(M\right)$$

where Embed(*) represents the embedding layer.

#### 2.1.3 Transformer decoder and attention matrix

In this study, the Transformer decoder is primarily used for the integration of gene expression information $G_{0}$ and molecular embedding information $M_{0}$. The decoder of the Transformer model is composed of *N* decoder layers. Each decoder layer consists of a masked multi-head self-attention layer, a multi-head attention layer, and a feed-forward neural network layer (Vaswani *et al.* 2017). Each layer is subjected to residual connections and layer normalization. Through N decoder layers, gene expression information $G_{0}$ and molecular embedding information $M_{0}$ are integrated into $V_{N}$.

The attention function is performed:

(17) $$\mathrm{Attention}\left(Q,K,V\right)=\mathrm{Softmax}\left(\frac{QK^{T}}{\sqrt{d_{K}}}\right)V$$

where $\mathrm{Softmax}\left(\frac{QK^{T}}{\sqrt{d_{k}}}\right)$ is the attention matrix; $d_{K}$ represents the dimension of the row vectors of the K matrix; and T indicates the matrix transpose.

The molecular embedding $V_{N-1}$ is updated to $V_{N-1}^{'}$ through the multi-head self-attention layer of the N-th decoding layer. Afterward, $V_{N-1}^{'}$ and gene expression information $G_{0}$ are integrated into $V_{N}^{'}$ through the multi-head attention layer of the N-th decoder layer.

(18) $$V_{N-1}^{'}=\;V_{N-1}\;+\;\mathrm{Attention}\left(V_{N-1},V_{N-1},V_{N-1}\right)$$

(19) $$V_{N}^{'}=\;V_{N-1}^{'}\;+\;\mathrm{Attention}\left(V_{N-1}^{'},G_{0},G_{0}\right)$$

Finally, $V_{N}^{'}$ is updated to $V_{N}$ through the feedforward neural network:

(20) $$V_{N}=\;V_{N}^{'}\;+\;\mathrm{FNN}\left(V_{N}^{'}\right)$$

#### 2.1.4 Generator and loss

The output $V_{N}$ of the transformer decoder is utilized by the generator to reconstruct molecules. The generator comprises a linear layer followed by a Softmax layer.

(21) $$\hat{M}=\mathrm{Softmax}(\mathrm{Linear}(V_{N}))$$

where $\hat{M}=({\hat{t}}_{1},\;{\hat{t}}_{2},\cdot \cdot \cdot ,{\;\hat{t}}_{L})$ represents the reconstructed molecules, Softmax(*) denotes the Softmax layer, and Linear(*) refers to the linear layer.

The objective of model training is to reconstruct the original molecule through the gene expression information of cell lines perturbed by this molecule. Therefore, the model's loss function is formulated as the Kullback–Leibler (KL) divergence between the reconstructed molecule $\hat{M}$ and the original molecule M.

(22) $$\mathrm{Loss}=KL(M,\hat{M})-\frac{1}{L}\sum_{i=1}^{L}t_{i}\;\text{log}\;\frac{t_{i}}{{\hat{t}}_{i}}$$

### 2.2 Model application

During the model application process, the input of the TransGEM model is only composed of two parts: the cell line type corresponding to a specific disease, and the gene expression difference values of 978 landmark genes between the normal tissue cells and this cell line. The above two pieces of information are encoded by the gene expression encoder and inputted to the decoder of the Transformer model. Finally, based on the output of the decoder, the generator generates a series of novel compound molecules.

## 3 Experiments

### 3.1 Dataset

The gene expression profiles are obtained from the LINCS1000 database, which are subjected to different perturbation conditions, including chemicals, gene knockouts, and overexpression (Subramanian *et al.* 2017). In this study, only gene expression data perturbed by compound treatments is considered. The LINCS1000 database comprises data of five levels, with level 3 data being utilized for model training and application, as opposed to the previously common usage of level 5 data in prior research. This is mainly because previous studies have shown that level 5 data exhibits high levels of noise when used to characterize gene expression profiles (Pham *et al.* 2022, Das *et al.* 2023, Pravalphruekul *et al.* 2023). The level 3 data of the LINCS1000 database represent the standardized and normalized results of experimentally measured fluorescence intensity values (Subramanian *et al.* 2017). Our study also indicates that under identical experimental conditions, the Pearson correlation coefficients for multiple replicate samples in level 3 data mostly exceed 0.7 (Supplementary Fig. S1), demonstrating excellent correlation among them. However, the Pearson correlation coefficients among replicate samples in the level 5 data mostly did not exceed 0.7 (Pham *et al.* 2021). Considering the influence of different compound doses and perturbation duration on the resulting gene expression data of cell lines, we selectively retained gene expression profiles perturbed by small molecule compounds administered at a dose of 10 μM with a perturbation duration of 24 h. In the LINCS 1000 database, the expression values of only 978 landmark genes were actually measured, while the expression values of the remaining 11 350 genes were inferred based on the expression values of these 978 landmark genes (Subramanian *et al.* 2017). In order to avoid excessive noise data, the expression values of only 978 landmark genes were retained in the gene expression profile for each sample. Due to the requirement of the model for gene expression changes of perturbed cell lines, the final data were the expression difference values of 978 landmark genes between each sample and its corresponding control sample. The expression difference values of 978 landmark genes for each sample were rounded to one decimal place. Finally, data of only the 14 cell lines (A375, A549, HA1E, HEK293, HELA, HEPG2, HT29, JURKAT, MCF10A, MCF7, MDAMB231, PC3, THP1, and YAPC) with the highest number of samples were retained. The data of the 14 cell lines were utilized to construct the subLINCS dataset. The basic and detailed statistical information on subLINCS datasets is shown in Table 1 and Supplementary Table S1.

**Table 1.** Basic information on subLINCS datasets.

Dataset	Molecule	Cell type	Training set	Validation set	Test set
subLINCS	6929	14	25 364	2000	14

### 3.2 Experiment settings

In the TransGEM model, the dimension of the hidden vectors of the Transformer decoder is set as 64. The layer number of the Transformer decoder and head number of multi-head attention are respectively set as 6 and 8. The dimension number of the feed-forward layer is set as 512. The training epoch, batch size, and learning rate are set as 200, 4, and 0.0001, respectively. All the above optimal parameters are from grid search.

### 3.3 Baselines

In this section, several previously established molecular generation models based on gene expression data are used as comparative baselines.

**CGAN.** The CGAN is operated by amalgamating Conditional GAN with a Wasserstein GAN augmented with a gradient penalty (Méndez-Lucio *et al.* 2020), which can generate inhibitor-like molecules through the gene-knockdown expression data.

**BiAAE.** The BiAAE is a Bidirectional Adversarial Autoencoder model designed for the generation of molecular structures in response to specified alterations in gene expression and vice versa (Shayakhmetov *et al.* 2020).

**FAME.** The FAME is a molecular graph generation model based on gene expression information (Pham *et al.* 2022).

**PaccMannRL.** The PaccMannRL is a composite model that combines a gene expression encoder with molecular decoder, incorporating reinforcement learning strategies (Born *et al.* 2021).

**BiCEV.** The BiCEV integrates both chemical language autoencoder and compound-expression autoencoder, enabling the generation of diverse molecules from gene expression data (Pravalphruekul *et al.* 2023).

**Gex2SGen.** The Gex2SGen is also a composite model consisting of a gene expression encoder and a molecular decoder. Gene expression VAE and molecular VAE are separately trained, followed by the integration of the gene expression encoder and molecular decoder into a new composite model (Das *et al.* 2023).

### 3.4 Evaluation metrics

For the evaluation of newly generated molecules, we consider the following five evaluation metrics:

Validity: The proportion of valid molecules in the generated molecules. The valid molecules SMILES transformed from SELFIES could be checked by RDKit (https://www.rdkit.org/).

Uniqueness: The proportion of nonrepetitive molecules in valid molecules.

Novelty: The proportion of molecules not in training set in valid molecules.

InDiv: The internal diversity (InDiv) of a group of molecules is evaluated according to the formula (Benhenda 2017).

$$\mathrm{IntDiv}\left(M\right)=1-\sqrt{\;\frac{1}{{\left|M\right|}^{2}}\sum_{m_{1},m_{2}\in M}\mathrm{smi}(m_{1},\;m_{2})\;}$$

where *M* indicates a group of molecules; InDiv (*M*) is the internal diversity of molecule group *M*; and m1 and m2 are the molecules in the molecular group *M*. The $\mathrm{smi}(m_{1},\;m_{2})$ represents the similarity of molecular fingerprints between m1 and m2. In this work, we choose RDKFingerprint and TanimotoSimilarity.

QED: Quantitative estimation of drug-likeness (QED) is the quantitative evaluation of drug-likeness degree of molecules (Bickerton *et al.* 2012).

SA: Synthetic accessibility (SA) score (Ertl and Schuffenhauer 2009) is the comprehensive score of molecular synthesis complexity.

### 3.5 Results

#### 3.5.1 Evaluation of four embedding forms of gene expression

**Figure 3.**
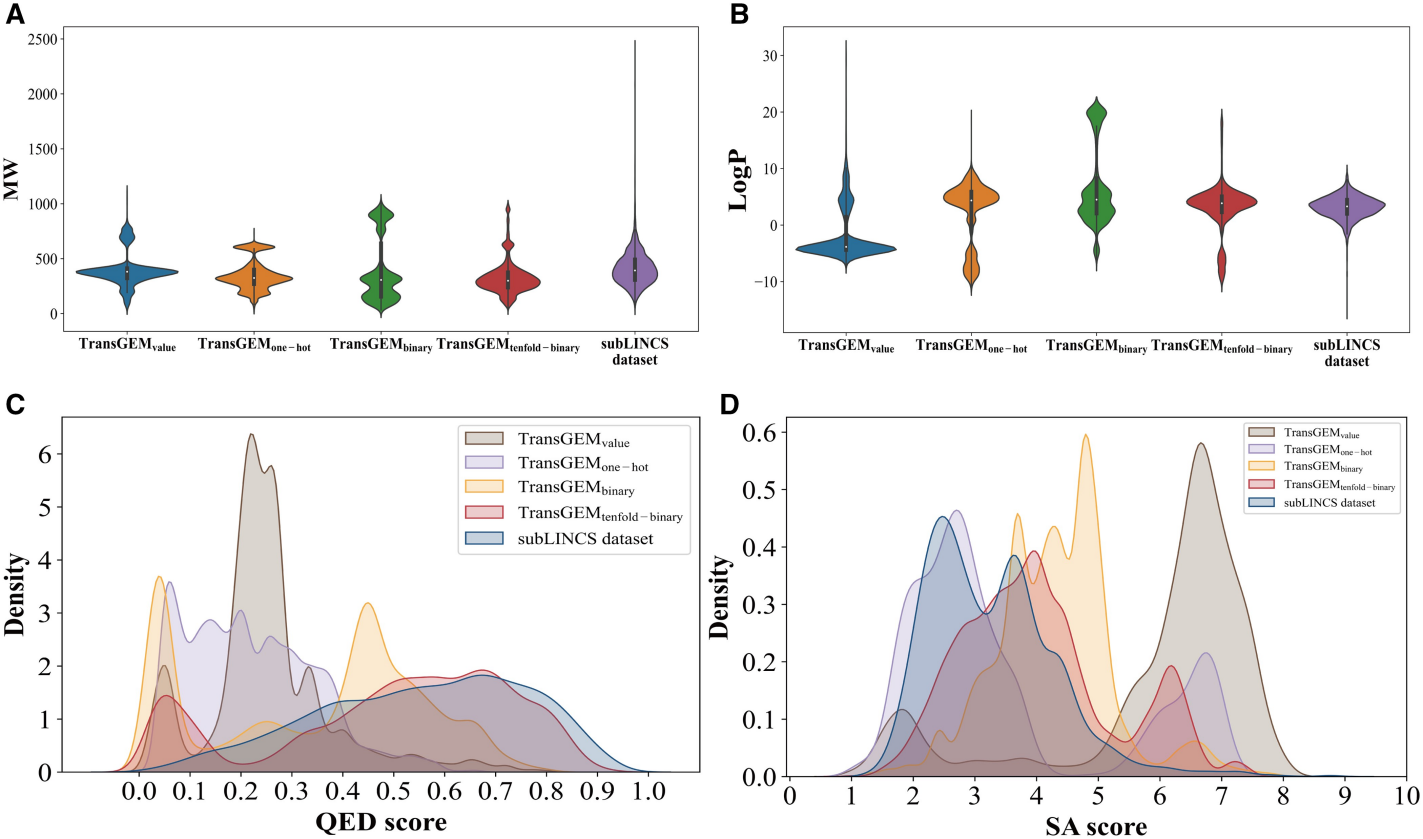
(A) Molecular weight, (B) LogP value, (C) QED value, and (D) SA score distributions of molecules generated by four TransGEM models and those molecules in the subLINCS dataset. Property distributions of molecules generated by the TransGEM_tenfold-binary_ model significantly outperforms the TransGEM models with the other three embedding forms

To evaluate applicability of four different embedding forms for gene expression difference values, TransGEM models based on four embedding forms (value, one-hot, binary, and 10-fold-binary) are defined as TransGEM_value_, TransGEM_one-hot_, TransGEM_binary_, and TransGEM_tenfold-binary_, respectively. The above-mentioned four TransGEM models are respectively trained, and the corresponding test results are presented in Table 2 and Fig. 3. The results show that molecules generated by all four TransGEM models exhibit outstanding validity and uniqueness, with molecular weights generally below 500 Da (Fig. 3A). However, the TransGEM_tenfold-binary_ model significantly outperforms the TransGEM models with the other three embedding forms in generating molecular uniquenesss and InDiv. Only the molecules generated by TransGEM_tenfold-binary_ model exhibits a close similarity in terms of the distribution of their LogP and QED values to the molecules in the subLINCS dataset (Fig. 3B and C). Moreover, a majority of the molecules generated by the TransGEM_tenfold-binary_ exhibits favorable SA scores (Fig. 3D).

**Table 2.** Evaluation of molecules generated by four TransGEM models.^a^

Model	Validity	Novelty	Uniqueness	InDiv
TransGEM_value_	99.9%	**100%**	59.9%	46.9%
TransGEM_one-hot_	**100%**	**100%**	54.0%	63.4%
TransGEM_binary_	**100%**	**100%**	63.6%	71.0%
TransGEM_tenfold-binary_	**100%**	**100%**	**84.9%**	**78.9%**

a The optimal results are shown in bold.

The above results indicate that, compared to the other three embedding forms, the 10-fold-binary embedding forms is more suitable for representing gene expression difference values. The potential reason is that directly inputting gene expression differentials into the model may lead to vector sparsity, resulting in the loss of significant differential information. The one-hot embedding form, while suitable for distinguishing between different categories, may not effectively quantify gene expression difference values. Both binary and 10-fold-binary embedding form can effectively quantify gene expression difference values, but compared to binary embedding form, 10-fold-binary embedding form has the capacity to magnify differences in gene expression, particularly emphasizing genes with substantial expression variations. Therefore, the TransGEM model based on the 10-fold-binary embedding form is adopted for the subsequent studies.

#### 3.5.2 Comparison with baseline model

Table 3 presents the performance of six baseline models, with evaluation results obtained from their respective studies. The results demonstrate that, in comparison to six baseline models, our model generates molecules with outstanding validity and uniqueness. While the uniqueness and internal diversity of the molecules generated by our model may not be optimal, they still exhibit exceptional performance. The PaccmannRL and BiCEV models can generate a high proportion of valid molecules due to the presence of a pre-trained compound decoder, which learns the structural rules of molecules (Das *et al.* 2023, Pravalphruekul *et al.* 2023). The FAME model represents molecules in the form of graphs, demonstrating outstanding validity in the generation of molecules.

**Table 3.** Evaluation of molecules generated by TransGEM and other baseline models.^a^

Model	Validity	Novelty	Uniqueness	InDiv
CGAN	8.5%			
BiAAE	76.0%			
FAME	83.8%	99.8%	86.6%	**86.7%**
PaccMannRL	84%–93%			
BiCEV	95.7%	**100%**	**98.2%**	77.0%
Gex2SGen	45%	88%	95%	
TransGEM	**100%**	**100%**	84.9%	78.9%

a The optimal results are shown in bold.

Our model uses a specialized gene expression embedding approach, distinguishing itself from other existing models that directly input gene expression profiles. Directly inputting gene expression values into a model can lead to the sparsification of the embedding matrix, resulting in the loss of substantial differential information. The gene expression embedding method applied by our model effectively quantifies gene expression values and mitigates the sparsity in the embedding matrix. Therefore, our model is capable of better capturing the intrinsic relationship between molecular structures and gene expression information, enabling it to generate molecules that are both valid and novel.

#### 3.5.3 Verification of attention matrix validity

**Figure 4.**
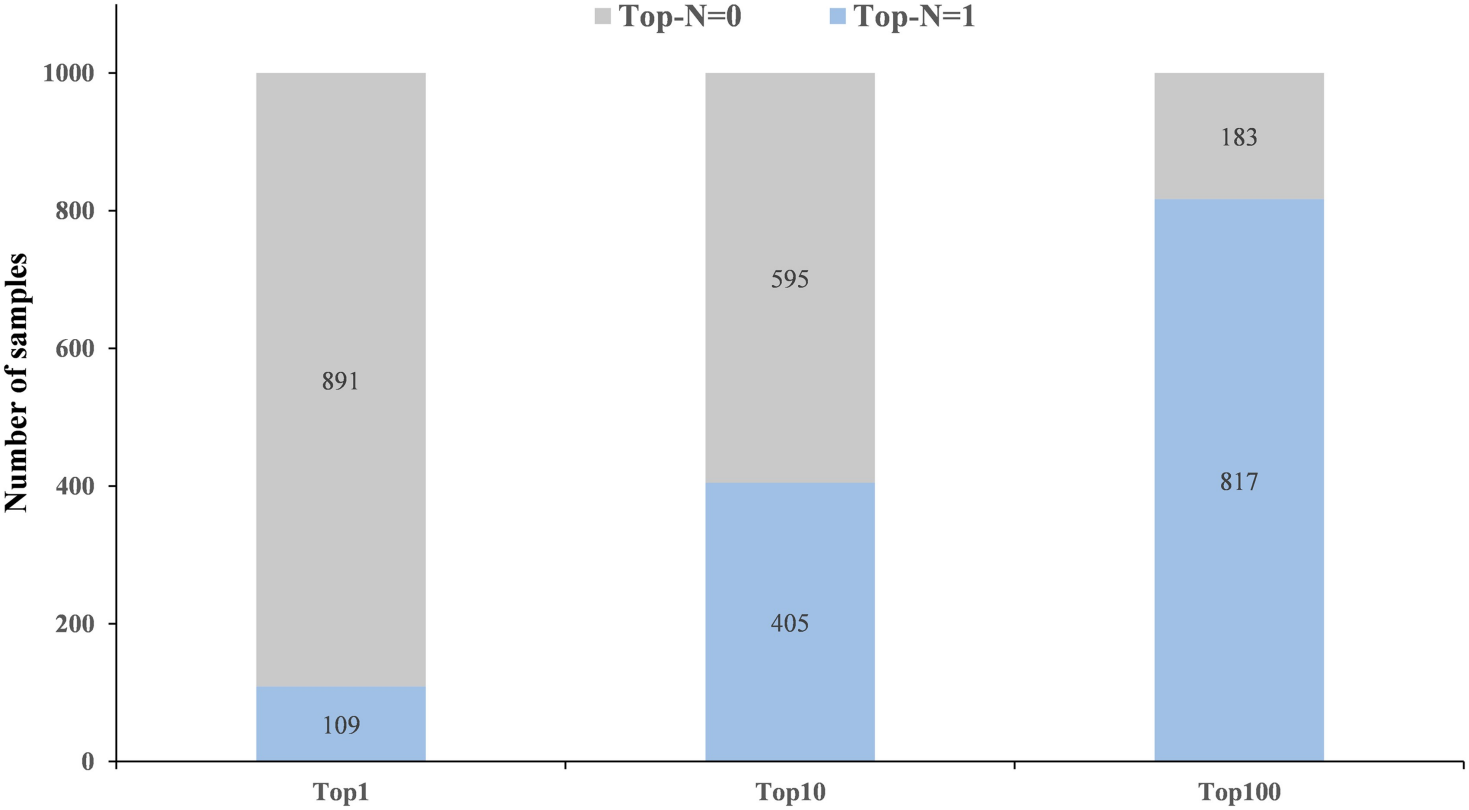
The score statistics of Top-*N*, where *N* = 10, 50, and 100. The gray color represents the number of “Top *N* = 0,” which means that among the top *N* genes ranked by attention scores, there are no genes that interact with the corresponding compounds in these samples; “Top *N* = 1” indicates the presence of genes interacting with the corresponding compounds of these samples among the top *N* genes ranked by attention scores.

The attention matrix is derived from the multi-head attention layer of the Transformer decoder, serving as a representation of the interaction information between gene expression data and molecule embeddings. When generating a particular molecule, the attention scores of 978 landmark genes can be computed through the attention matrix. To validate the relevance of genes with high attention scores to the generation of a particular molecule, we conduct the following study. We select 1000 samples from the subLINCS dataset and utilize the TransGEM model to reconstruct corresponding molecules based on the gene expression information of these samples. Subsequently, we extract attention matrices for each sample and computed attention scores for 978 landmark genes based on their respective attention matrices. We establish a performance metric named Top-*N* to assess whether there are genes that interact with molecules corresponding to the sample among the top *N* genes ranked by attention scores for a given sample. If, during the generation of molecule of a particular sample from the gene expression information of this sample, there are genes within the top *N* attention-ranked genes that interact with this molecule, then Top-*N* = 1, otherwise, Top-*N* = 0. The interaction information between molecules and genes is sourced from the Therapeutic Target database (Chen *et al.* 2002). Figure 4 illustrates that the number of samples with a Top-10 score of 1 is over 100, and upon scaling N to 100, the proportion of samples with a Top-100 score of 1 exceeds 80%. This indicates that genes with higher attention scores during molecule generation are more likely to interact with the molecule, thereby validating the attention matrix obtained by the TransGEM model decoder is valid.

#### 3.5.4 Case study

**Figure 5.**
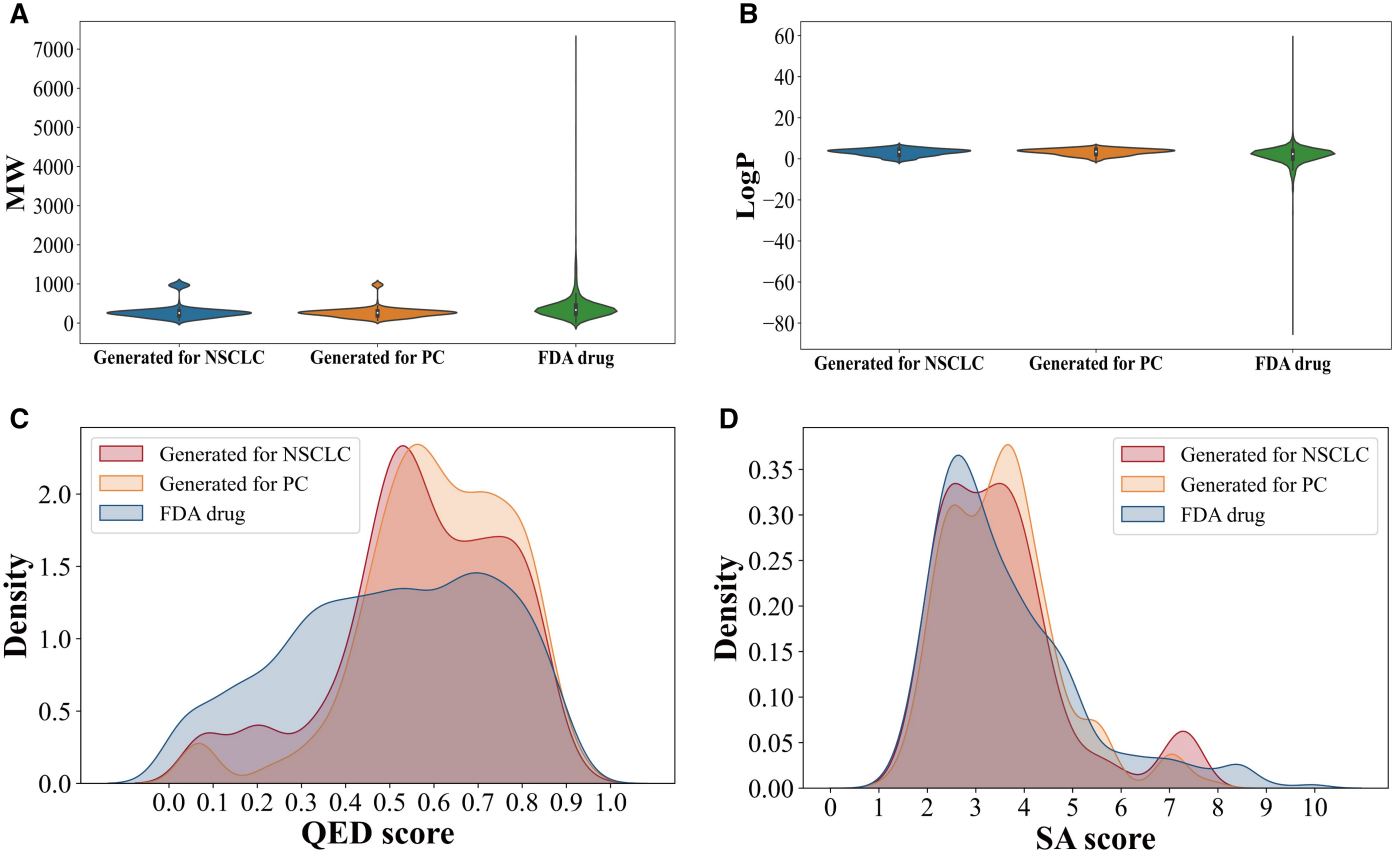
(A) Molecular weight, (B) LogP value, (C) QED value, and (D) SA score distributions of molecules generated by TransGEM model targeting PC and FDA-approved drugs. Property distributions of molecules generated by TransGEM are largely consistent with those of FDA-approved drugs

In order to further validate whether the TransGEM model can generate molecules with potential bioactivity, the model is used for case studies of NSCLC and PC. The cell lines corresponding to NSCLC and PC are designated as A549 and PC3, respectively. For each disease, 1000 molecules are generated and evaluated (Fig. 5 and Table 4). The results indicate that the TransGEM model is still able to generate 100% valid molecules. Both uniqueness and InDiv of the molecules generated by our TransGEM model reach above 80%. Furthermore, the distributions of molecular weight and LogP value of the generated molecules targeting the two diseases are largely consistent with those of FDA-approved drugs (Food and Drug Administration). Moreover, the TransGEM model can also generate molecules with high drug-likeness or low synthetic complexity targeting these two diseases (Fig. 5C and D).

**Table 4.** Evaluation of molecules generated by TransGEM targeting PC and NSCLC.

Disease	Validity	Uniqueness	InDiv
PC	100%	81.2%	80.7%
NSCLC	100%	82.9%	81.3%

To further validate whether the TransGEM model can generate molecules with potential biological activity, molecule docking is performed between the molecules generated for NSCLC and PC and the known targets of the two diseases. According to Section 3.5.2, it is evident that, in the generation of a particular molecule, the higher the attention score of a gene, the more likely it is to interact with that molecule. Therefore, for known disease target genes, molecules with the top 200 attention ranking of this target gene during the molecular generation process are selected to construct a molecular library. This library is used for molecular docking simulations with the corresponding target proteins associated with this target gene. The results presented in this section are exclusively related to PC, while the results pertinent to NSCLC can be found in Supplementary material.

**Figure 6.**
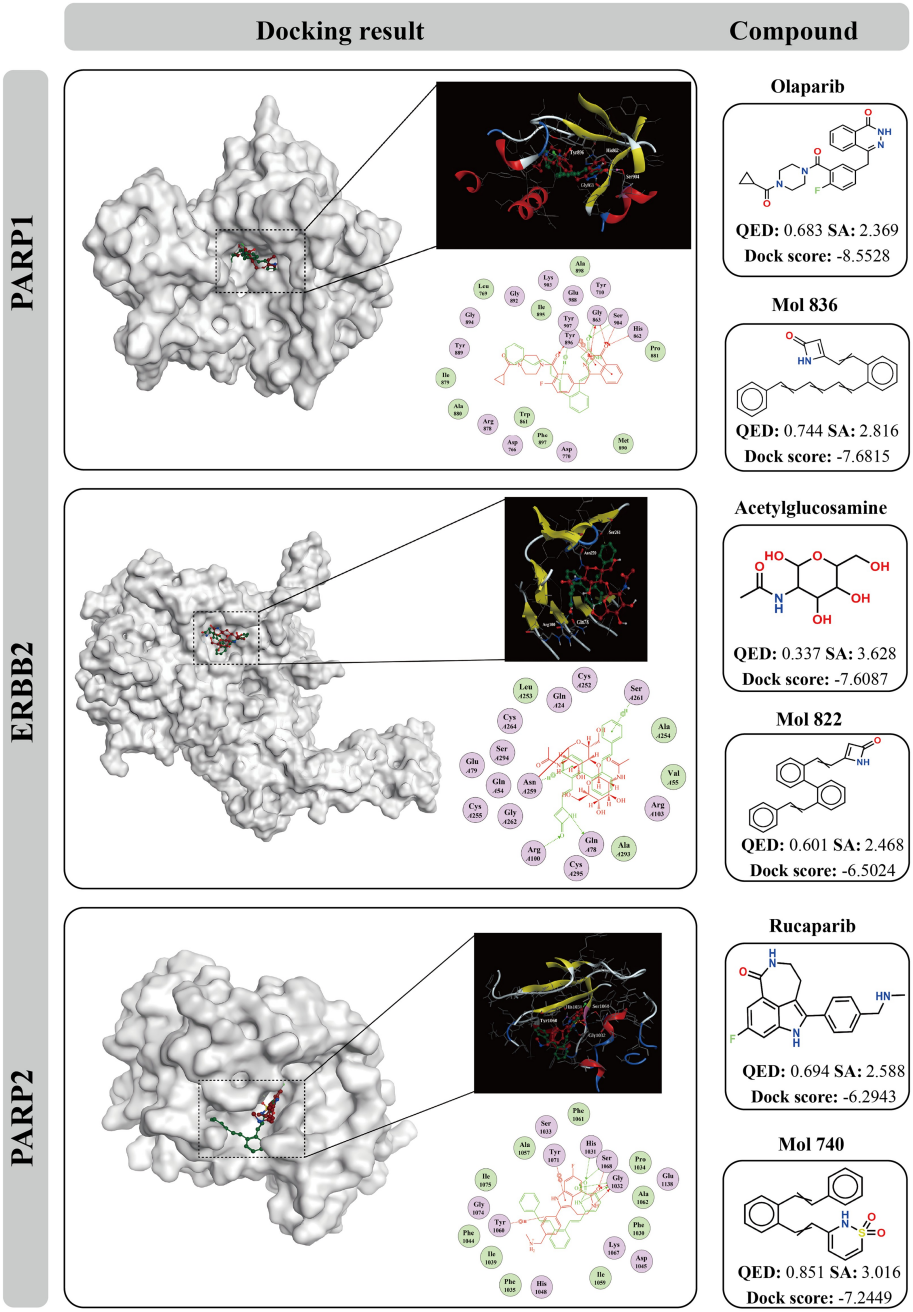
The docking analysis of PARP1 with Olaparib and Mol836; ERBB2 with Acetylglucosamine and Mol822; and PARP2 with Rucaparib and Mol740. The docking score, QED score, SA score and 2D structure of each molecule were attached to the corresponding binding mode diagram.

AS for PC, the three known drug targets, poly ADP-ribose polymerase 1 (PARP1) (Deshmukh and Qiu 2015), poly ADP-ribose polymerase 2 (PARP2) (Gui *et al.* 2019), and erb-b2 receptor tyrosine kinase 2 (ERBB2) (Yumoto *et al.* 2023), are selected for inclusion in this study. The crystal structure of the PARP1, PARP2, and ERBB2 are downloaded from the PDB database (Burley *et al.* 2021), with a PDB ID of 7kk4 (Ryan *et al.* 2021), 4bjc (Haikarainen *et al.* 2014), and 5my6 (D’Huyvetter *et al.* 2017), respectively. The 1000 molecules with top 200 attention ranking of *PARP1* are screened from the generated molecules. For *PARP2* and *ERRB2*, the numbers of screened molecules are 581 and 866, respectively. Three sets of molecules are used to construct a molecular library, each subjected to molecule docking simulations with their respective target proteins. Figure 6 illustrates the optimal docking results alongside their corresponding molecular structures. The results indicate that Mol836 exhibits docking scores with PARP1 comparable to those of Olaparib. Olaparib serves as the corresponding ligand in the crystal structure of PARP1 and is an FDA-approved drug for the treatment of PC (Deeks 2015). A similar scenario arises between Mol822 and Acetylglucosamine. Excitingly, the docking score of Mol704 with PARP2 even surpasses that of Rucaparib with PARP2. Furthermore, the QED scores of Mol836, Mol822, and Mol704 are superior to those of Olaparib, Acetylglucosamine, and Rucaparib, respectively. Furthermore, as depicted in Fig. 6, the binding mode diagrams of molecules with target proteins also indicate that the binding patterns of molecules generated by the TransGEM model are similar to the binding modes of the original ligands from the crystal structures of target proteins. For instance, both Mol836 and Olaparib exhibit the capability to form stable hydrogen bond interactions with Gly863 and Ser904 residues of PARP1. Previous research has demonstrated that Olaparib forms stable hydrogen bond with the His862, Gly863, Tyr896, and Ser904 residues of the PARP1 protein, of which Gly863 and Ser904 are the key active amino acids (Ryan *et al.* 2021). The analogous scenario is observed between Mol822 and Acetylglucosamine, as well as Mol704 and Rucaparib. The above results indicate that the TransGEM model possesses the potential to generate molecules with potential biological activity.

During the process of generating molecules targeting PC, the top 10 genes with the highest attention scores are collected from the TransGEM model (Table 5). Surprisingly, all the 10 genes are found to be associated with the onset of PC. For instance, the *ANXA7* encodes membrane-associated protein A7, which inhibits PC metastasis by activating GTPase (Liu *et al.* 2018). The *HLA-DRA* product, leukocyte antigen presentation, exhibits significantly reduced expression in immune cells of PC patients (Vuk-Pavlović *et al.* 2010). *DECR1* encodes a testosterone-suppressed survival factor that prevents the accumulation of unsaturated fatty acid oxidation, safeguarding prostate tumor cells from ferroptosis (Nassar *et al.* 2020). The precursor protein encoded by the *APP* promotes PC tumor growth (Takayama *et al.* 2009). *GPC1*, encoding phosphatidylinositol glycan anchor biosynthesis class-1, is considered a reliable PC marker (Levin *et al.* 2018). PARP1, encoded by the *PARP1*, is one of the targets currently used for PC treatment (Deshmukh and Qiu 2015). The product of the *ELOVL6*, associated with lipid metabolism in the human body, exerts an anti-PC effect through the inhibition of its activity (Zhang *et al.* 2023). DNA methyltransferase 3A, encoded by *DNMT3A*, inhibits PC by regulating autophagy in PC cells (Zhang *et al.* 2021). *CCND1*, encoding cyclin D1, is involved in regulating PC cell proliferation and the cell cycle process (Long *et al.* 2018). The *DDB2* expresses at lower levels in PC tissue, potentially disrupting androgen homeostasis and inducing subsequent PC growth (Gilson *et al.* 2019). This indicates that when generating molecules targeting a particular disease, the TransGEM model indeed pays more attention to genes that are more closely associated with the onset of the disease.

**Table 5.** Top 10 genes with high attention ranking corresponding to generated molecule targeting PC.

Attention rank	Gene name	References
1	*ANXA7*	Liu *et al.* (2018)
2	*HLA-DRA*	Vuk-Pavlović *et al.* (2010)
3	*DECR1*	Nassar *et al.* (2020)
4	*APP*	Takayama *et al.* (2009)
5	*GPC1*	Levin *et al.* (2018)
6	*PARP1*	Deshmukh and Qiu (2015)
7	*ELOVL6*	Zhang *et al.* (2023)
8	*DNMT3A*	Zhang *et al.* (2021)
9	*CCND1*	Long *et al.* (2018)
10	*DDB2*	Gilson *et al.* (2019)

The above results demonstrate that the TransGEM model performs well when it is applied to specific diseases. TransGEM model is capable of generating structurally novel molecules with high drug-likeness and low synthetic complexity, and the generated molecules can stably interact with the active amino acid sites of disease targets. This suggests that TransGEM model can generate molecules with potential bioactivity. When generating molecules targeting a particular disease, the model allocates high attention to genes closely associated with the onset of the disease. These genes have the potential to serve as therapeutic targets for the disease and deserve further investigation.

## 4 Conclusion

This study constructs the TransGEM model, and this model adopts a specialized gene expression encoder to better embed gene expression difference values. The performance of the TransGEM model surpasses that of the baseline model, and can generate molecules with desirable evaluation metrics and property distributions. TransGEM model is applied to case study of PC and NSCLC in this study. The case study shows that this model can generate structurally novel molecules that exhibit favorable properties and form stable interactions with important active sites of known disease targets. Therefore, the TransGEM model has great potential to generate biologically active molecules. In addition, the genes with high attention scores obtained from the TransGEM model are mostly related to the onset of the disease, and these genes have the potential to become therapeutic targets for the disease, which need to be further investigated.

However, our model still has some limitations. In this study, only the 978 gene expression difference values are utilized to characterize the expression difference between disease cells and normal tissue cells, which inevitably leads to the loss of a substantial amount of differential information. Future studies are suggested to utilize genome-wide differential expression information in the TransGEM model, which will further enhance its performance.

## Supplementary Material

btae189_Supplementary_Data
