# Supplementary material for: TransGEM: a molecule generation model based on Transformer with gene expression data
Source: Bioinformatics. 2024 Apr 17;40(5):btae189. doi: 10.1093/bioinformatics/btae189 (PMC11078772; doi:10.1093/bioinformatics/btae189)
Supplement: btae189_Supplementary_Data [file btae189_supplementary_data.docx]

**Supporting Information**

**Data processing**

The Library of Integrated Network-based Cellular Signatures 1000 landmark genes (LINCS1000) database comprises gene expression data at five levels of various perturbed cell lines. The level 3 data is utilized for model training and application, as opposed to the previously common usage of level 5 data in prior research. We selectively retained gene expression profiles perturbed by small molecule compounds administered at a dose of 10μM with a perturbation duration of 24 hours. Due to the larger sample sizes in these two experimental conditions compared to others. We assigned labels based on 'cell line + compound' and identified duplicate samples within the same label. The Average Pearson Correlation (APC) score between duplicate samples under the same label was computed, and samples with an APC score below a threshold were deemed unreliable. In our setup, this threshold was set at 0.7. The density function distribution of APC scores is illustrated in Supplementary Figure S1. Samples with APC scores below the threshold within the same label, as well as labels with fewer than 2 samples, were filtered out. The remaining multiple gene expression profiles under the same label were median-aggregated into one label sample. Finally, only the gene expression data corresponding to the 14 cell lines with the highest number of label samples were retained, forming the subLINCS dataset.

**Spatial distribution of molecules**

We randomly selected 100,000 molecules from the PubChem database as a random molecule set and collected two sets of FDA-approved drugs related to NSCLC and PC. The random molecule set, generated molecules, and drugs were all transformed into circular (ECFP6) fingerprints. Subsequently, the molecular fingerprints were visualized through Principal Component Analysis (PCA) dimensionality reduction (Figure S2 and S3). The results indicate that, compared to the random molecule set, molecules generated for NSCLC and PC predominantly concentrate in a specific region. This suggests that the process of generating molecules by the model is not random but rather based on specific gene expression information. Furthermore, the distribution region of generated molecules overlaps with the distribution region of existing drugs for the respective diseases. This implies that the TransGEM model can generate molecules structurally similar to existing therapeutic drugs for the diseases.

**Docking results analysis of molecules generated for Non-Small Cell Lung Cancer (NSCLC)**

AS for NSCLC, the 3 known drug targets, epidermal growth factor receptor (EGFR) (Stewart *et al.*, 2015); LYN proto-oncogene, Src family tyrosine kinase (LYN) (Kim *et al.*, 2014) and SRC proto-oncogene, non-receptor tyrosine kinase (SRC) (Giaccone and Zucali, 2008), are selected for inclusion in this study. The crystal structure of the EGFR, LYN and SRC are downloaded from the PDB database (Burley *et al.*, 2021), with a PDB ID of 4i24 (Gajiwala *et al.*, 2013), 5xy1(Kinoshita *et al.*, 2006), and 4f5b (Kaneko *et al.*, 2012), respectively. The 9 molecules he top 200 attention ranking of *EGFR* are screened from the generated molecules. For *LYN* and *SRC*, the numbers of screened molecules are 728 and 788, respectively. Three sets of molecules are employed to construct a molecular library, each subjected to molecular docking simulations with their respective target proteins. Figure S4 illustrates the optimal docking results alongside their corresponding molecular structures. The results indicate that Mol538 exhibits docking score with EGFR comparable to this of Dacomitinib. Dacomitinib serves as the corresponding ligand in the crystal structure of EGFR and is an FDA-approved drug for the treatment of PC (Deeks, 2015). A similar scenario arises between Mol242 and PubChem78210204, as well as Mol745 and O-Phospho-L-tyrosine. Furthermore, the QED scores of Mol538, Mol242, and Mol745 are superior to those of Dacomitinib, PubChem78210204, and O-Phospho-L-tyrosine, respectively. Furthermore, as depicted in Figure S4, the binding mode diagrams of molecules with target proteins also indicate that the binding patterns of molecules generated by the TransGEM model are similar to the binding modes of the original ligands from the crystal structures of target proteins. For instance, both Mol538 and Dacomitinib exhibit the capability to form stable hydrogen bond interactions with Gly863 residues of PARP1. Relevant studies have indicated that Olaparib form stable hydrogen bonds with Gln791, Met793, Cys797, Asp800, and Asp855 residues of the EGFR protein, of which Cys797 is the key active amino acids(Gajiwala *et al.*, 2013). The analogous scenario is observed between Mol242 and PubChem78210204, as well as Mol745 and O-Phospho-L-tyrosine. The above results indicate that the TransGEM model possesses the potential to generate molecules with potential biological activity.

**Attention matrix** **analysis of molecules generated for NSCLC**

When generating molecules for NSCLC, eight out of the top 10 genes in the attention ranking have known associations with the occurrence of the disease. For instance, the elevated expression of the *MEST* in NSCLC cells may be correlated with tumor initiation and malignant transformation (Nakanishi *et al.*, 2004). *DDX10* promotes human lung cancer proliferation by influencing U3 small nucleolar ribonucleoprotein 4 (Liu *et al.*, 2021). The product of the *DDB2* enhances DNA damage response, increasing the radioresistance of NSCLC cells (Zou *et al.*, 2016). The glutaredoxin encoded by *GLRX* plays a crucial role in oxidative stress, and its reduced activity promotes apoptosis and cell cycle arrest in NSCLC cells (Wang *et al.*, 2019). The antisense RNA of *CBR3* reduces the proliferation, invasion, and migration of NSCLC cells, induces apoptosis, and enhances radio-sensitivity (Liu et al., 2022). Fusion of the *ATP1B1* with the *NRG1* disrupts the activity of NSCLC cells (Laskin *et al.*, 2020). The biliverdin reductase A encoded by *BLVRA* produces bilirubin to maintain intracellular redox homeostasis, further reducing the drug resistance of NSCLC (Xu *et al.*, 2014). The expression of the *HOXA10* promotes malignant proliferation and radioresistance of NSCLC cells (Zhu *et al.*, 2022). These results indicate that when the TransGEM model generates molecules specific to a particular disease, the genes with higher attention rankings are indeed associated with the occurrence of the disease, suggesting their potential as therapeutic targets for the disease.


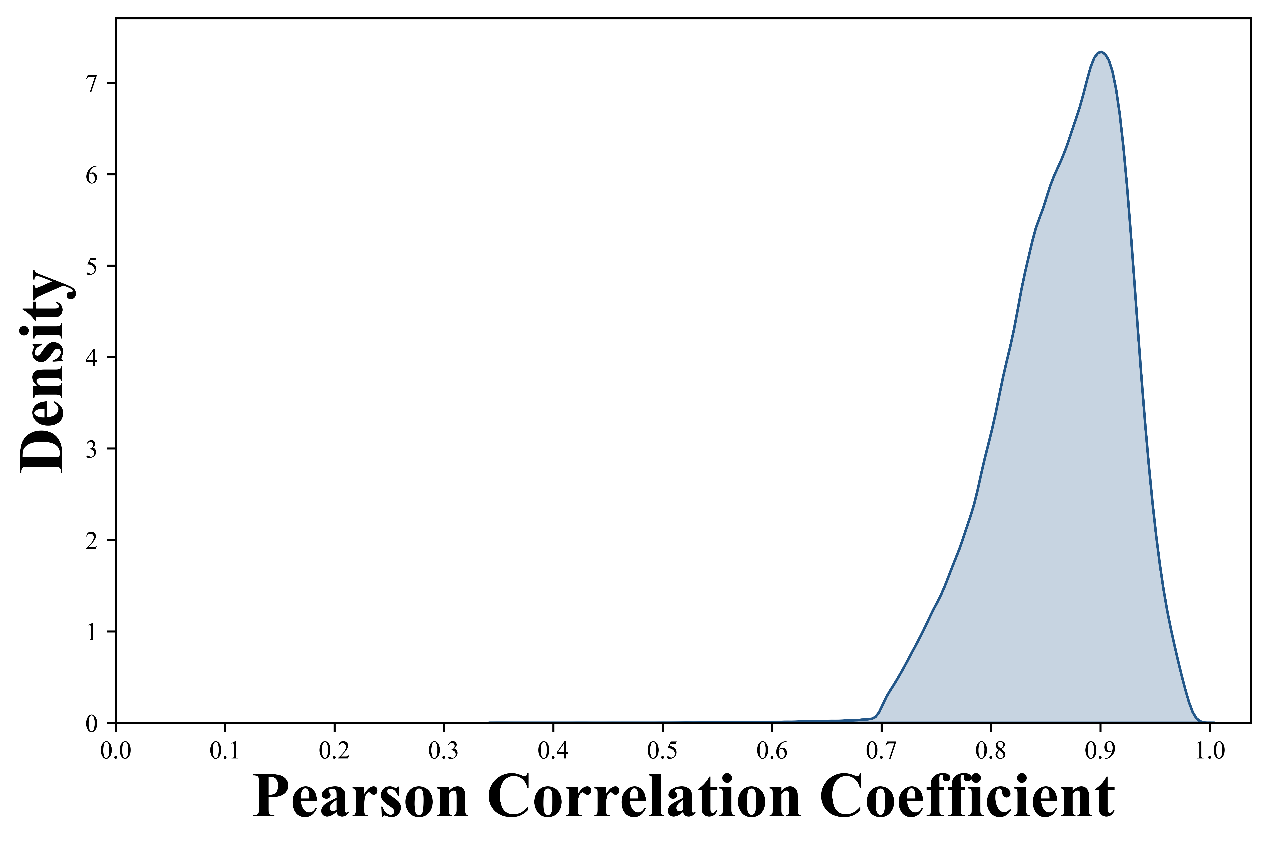


**Figure S1.** The density function of APC score for level 3 data.


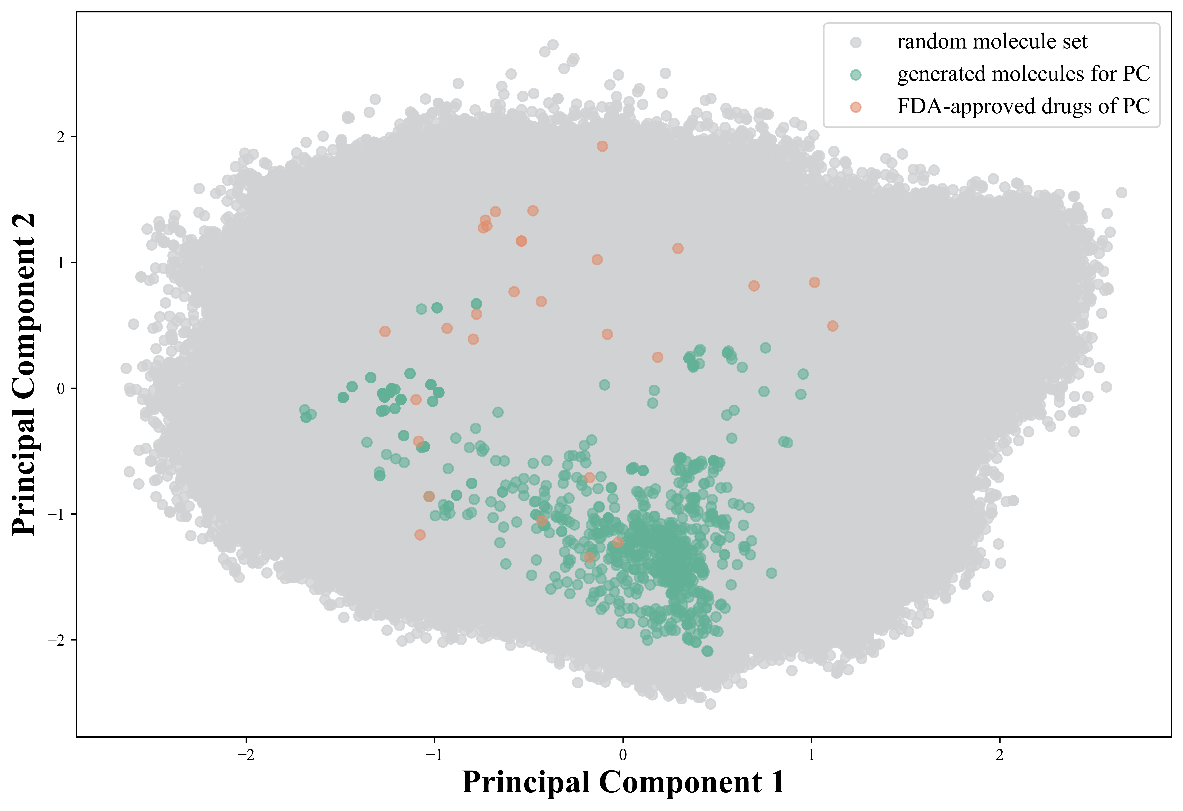


**Figure S2.** Visualization of random molecule set, generated molecules, and FDA-approved drugs related PC.


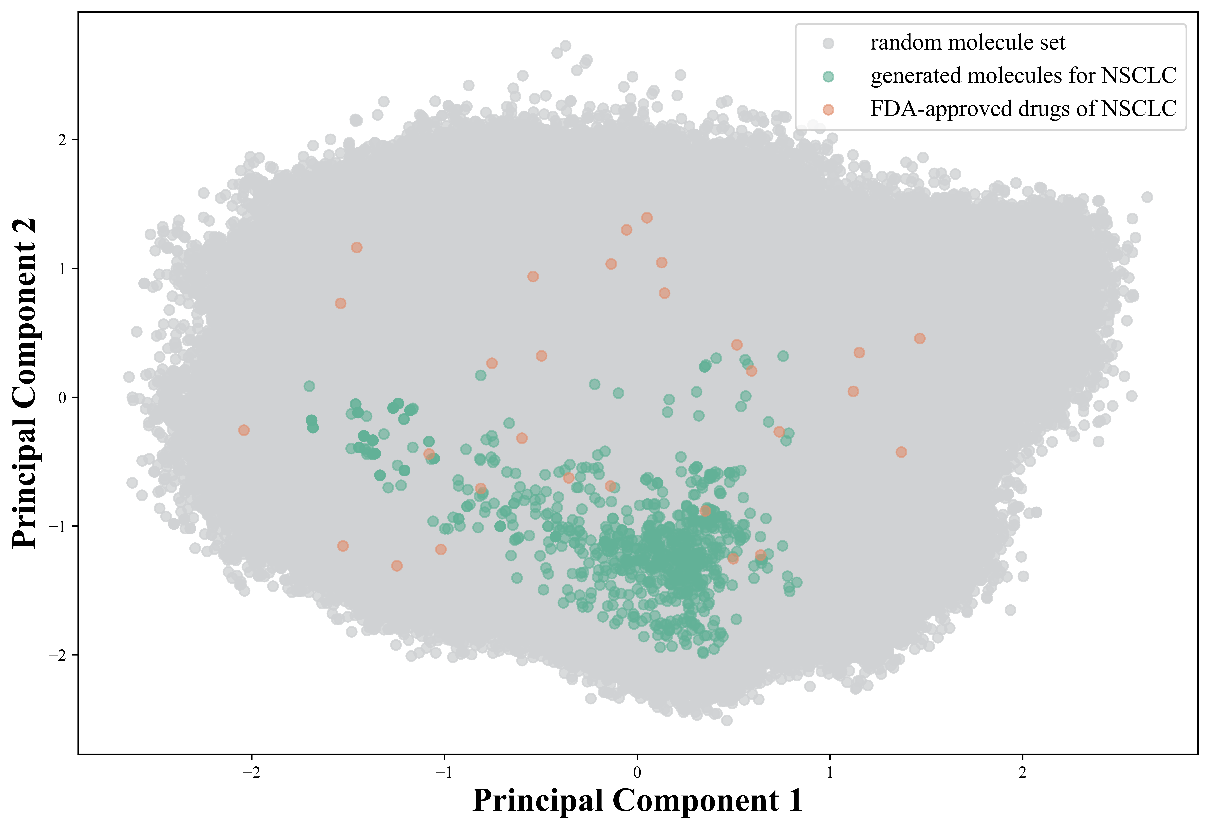


**Figure S3.** Visualization of random molecule set, generated molecules, and FDA-approved drugs related NSCLC.


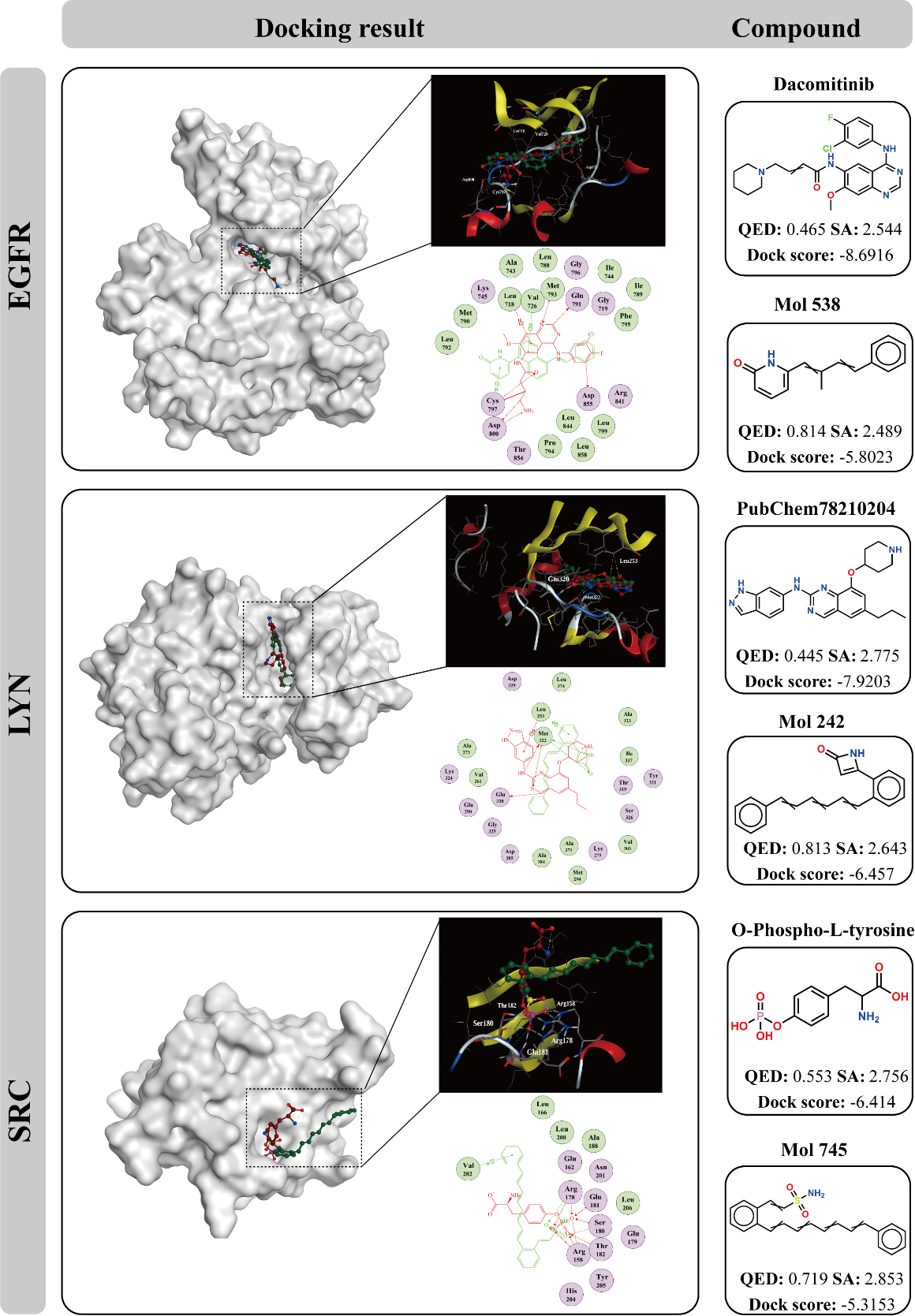


**Figure S4.** The docking analysis of EGFR with Dacomitinib (red ball-and-stick) and Mol538 (green ball-and-stick); LYN with Pubchem78210204 (red ball-and-stick) and Mol242 (green ball-and-stick); and PARP2 with O-Phospho-L-tyrosine (red ball-and-stick) and Mol745 (green ball-and-stick). The docking score, QED score, SA score and 2D structure of each molecule were attached to the corresponding binding mode diagram.

**Table S1.** Detailed information on subLINCS dataset.

| **Cell type** | **Molecule** | **Training set** | **Validation set** | **Test set** |
| --- | --- | --- | --- | --- |
| **PC3** | 4890 | 4531 | 358 | 1 |
| **MCF7** | 4328 | 4010 | 317 | 1 |
| **HA1E** | 3402 | 3152 | 249 | 1 |
| **A375** | 3353 | 3107 | 245 | 1 |
| **HT29** | 2973 | 2754 | 218 | 1 |
| **A549** | 2380 | 2205 | 174 | 1 |
| **HELA** | 1337 | 1238 | 98 | 1 |
| **YAPC** | 1170 | 1084 | 85 | 1 |
| **HEPG2** | 839 | 777 | 61 | 1 |
| **MCF10A** | 744 | 689 | 54 | 1 |
| **MDAMB231** | 608 | 563 | 44 | 1 |
| **HEK239** | 595 | 551 | 43 | 1 |
| **THP1** | 394 | 365 | 28 | 1 |
| **JURKAT** | 365 | 338 | 26 | 1 |

**Table S2.** Top 10 genes with high attention ranking corresponding to generated molecule targeting NSCLC.

| Attention rank | Gene name | References |
| --- | --- | --- |
| 1 | *MEST* | (Nakanishi *et al.*, 2004) |
| 2 | *DDX10* | (Liu *et al.*, 2021) |
| 3 | *DDB2* | (Zou *et al.*, 2016) |
| 4 | *GLRX* | (Wang *et al.*, 2019) |
| 5 | *CBR3* | (Liu *et al.*, 2022) |
| 6 | *CRYZ* |  |
| 7 | *ATP1B1* | (Laskin *et al.*, 2020) |
| 8 | *BLVRA* | (Xu *et al.*, 2014) |
| 9 | *PHKA1* |  |
| 10 | *HOXA10* | (Zhu *et al.*, 2022) |

**References**

Burley,S.K. *et al.* (2021) RCSB Protein Data Bank: powerful new tools for exploring 3D structures of biological macromolecules for basic and applied research and education in fundamental biology, biomedicine, biotechnology, bioengineering and energy sciences. *Nucleic Acids Research*, **49**, D437–D451.

Deeks,E.D. (2015) Olaparib: first global approval. *Drugs*, **75**, 231–240.

Gajiwala,K.S. *et al.* (2013) Insights into the Aberrant Activity of Mutant EGFR Kinase Domain and Drug Recognition. *Structure*, **21**, 209–219.

Giaccone,G. and Zucali,P.A. (2008) Src as a potential therapeutic target in non-small-cell lung cancer. *Annals of Oncology*, **19**, 1219–1223.

Kaneko,T. *et al.* (2012) Superbinder SH2 Domains Act as Antagonists of Cell Signaling. *Science Signaling*, **5**, ra68–ra68.

Kim,Y. *et al.* (2014) LYN is a new prognostic and therapeutic target in non-small cell lung cancer. *CANCER RESEARCH*, **74**.

Kinoshita,T. *et al.* (2006) Structure of human Fyn kinase domain complexed with staurosporine. *Biochemical and Biophysical Research Communications*, **346**, 840–844.

Laskin,J. *et al.* (2020) NRG1 fusion-driven tumors: biology, detection, and the therapeutic role of afatinib and other ErbB-targeting agents. *Ann Oncol*, **31**, 1693–1703.

Liu,C. *et al.* (2021) DDX10 promotes human lung carcinoma proliferation by U3 small nucleolar ribonucleoprotein IMP4. *Thorac Cancer*, **12**, 1873–1880.

Liu,S. *et al.* (2022) Long noncoding RNA CBR3-AS1 mediates tumorigenesis and radiosensitivity of non-small cell lung cancer through redox and DNA repair by CBR3-AS1 /miR-409-3p/SOD1 axis. *Cancer Letters*, **526**, 1–11.

Nakanishi,H. *et al.* (2004) Loss of imprinting of PEG1/MEST in lung cancer cell lines. *Oncol Rep*, **12**, 1273–1278.

Stewart,E.L. *et al.* (2015) Known and putative mechanisms of resistance to EGFR targeted therapies in NSCLC patients with EGFR mutations—a review. *Transl Lung Cancer Res*, **4**, 67–81.

Wang,L. *et al.* (2019) GLRX inhibition enhances the effects of geftinib in EGFR-TKI-resistant NSCLC cells through FoxM1 signaling pathway. *J Cancer Res Clin Oncol*, **145**, 861–872.

Xu,Y. *et al.* (2014) Genetic polymorphisms in oxidative stress-related genes are associated with clinical outcome in patients with advanced non-small cell lung cancer receiving tyrosine kinase inhibitors. *Am J Cancer Res*, **4**, 934–942.

Zhu,H. *et al.* (2022) Circ_0010235 Regulates HOXA10 Expression to Promote Malignant Phenotypes and Radioresistance in Non-small Cell Lung Cancer Cells Via Decoying miR-588. *Balkan Med J*, **39**, 255–266.

Zou,N. *et al.* (2016) DDB2 increases radioresistance of NSCLC cells by enhancing DNA damage responses. *Tumour Biol*, **37**, 14183–14191.
